# Quality of measuring devices enclosed with paediatric oral liquid dosage forms of medicines registered in Sri Lanka

**DOI:** 10.1371/journal.pone.0294690

**Published:** 2023-11-22

**Authors:** Abarna Nadeshkumar, Gitanjali Sathiadas, Shalini Sri Ranganathan

**Affiliations:** 1 Faculty of Allied Health Sciences, Department of Pharmacy and Pharmaceutical Sciences, University of Sri Jayewardenepura, Nugegoda, Sri Lanka; 2 Faculty of Medicine, Department of Pharmacology, University of Colombo, Colombo, Sri Lanka; 3 Faculty of Medicine, Department of Paediatrics, University of Jaffna, Jaffna, Sri Lanka; Siksha O Anusandhan University School of Pharmaceutical Sciences, INDIA

## Abstract

**Introduction:**

Oral liquid dosage forms remain popular in several middle income countries. The accuracy of liquid dosage form dosing depends on the accuracy and availability of measuring devices. Lack of quality oral liquid measuring devices will lead to medication errors. Hence there is an urgent need to describe the quality of manufacturer supplied measuring devices enclosed with paediatric oral liquid dosage forms currently registered in Sri Lanka.

**Methodology:**

Standards for measuring devices were developed after a detailed literature search. Multidisciplinary panel rated each standard for the necessity criteria on a 9 point Likert scale. Standards with overall panel median score of ≥ 7 with agreement were selected. A cross-sectional study was done. All the measuring devices, labels and instructions enclosed with the registered products were assessed against the standards developed. Three volumes of liquid antibacterials were measured using the enclosed measuring device. Accuracy of the volumes was measured.

**Results:**

Of the total products (n = 202) only 126 were packed with a dosing device. Around quarter of the oral liquid dosage forms (n = 36) did not have a measuring device. More than half of the measuring devices aligned with all the standards developed. Out of 44 oral liquid paediatric antimicrobials measuring cups (n = 25, 56.8%, 95% CI: 41%-72%) were enclosed more and less error was seen with measuring cups.

**Conclusion:**

The quality of oral liquid measuring devices were not satisfactory. Quality could be further improved if the regulatory body request the manufactures/importers to adhere to the standards developed. Correct volumes were not measured using the measuring devices provided with the liquid antimicrobial agents

## Introduction

Oral liquid dosage form is the most common dosage form prescribed for children in several low to middle income countries (LMICs), despite a paradigm shift in the choice of paediatric dosage forms [1].

Oral liquid dosage forms have some unique advantages over other oral dosage forms such as dose flexibility, ease of administration, familiarity, wide availability and relatively less expensive than the new paediatric oral dosage forms, such as oro-dispersible tablets. Though there is a change in the preferred oral dosage forms for young children to dispersible tablets, oral liquid dosage forms continue to be popular in many LMICs. Use of oral liquid dosage forms for children will remain for a long time in LMICs like Sri Lanka. Significant limitation identified for liquid dosage form is the difficulty in accurately measuring required volume [2]. Caregivers use assorted devices such as measuring cups, measuring spoons, droppers, oral syringes and household spoons to measure the required volume of liquid dosage form. Accurate volume measurement with measuring devices is dependent on instructions, labelling, quality of measuring device, knowledge and prior experience in measuring. Devices supplied by the manufacturers appear to be better than the domestic devices. Incorrect measuring devices were one of the frequent sources of medication overdoses [3]. Though manufacturers usually include the measuring device, medication errors are known to occur due to lack of standardization of labelling and measuring devices [4]. The United States Food and Drug Administration (US FDA) published the guidelines for the measuring devices packaged in the over the counter liquid medications for industries in 2011 [5]. Such standards for measuring devices are not available in Sri Lanka.

Antimicrobials use has increased globally [6]. Getting an adequate concentration of antimicrobial agents at the site of infection is crucial in preventing selection of drug resistance mutants. In Sri Lanka, oral antimicrobial agents are most commonly given as liquid dosage form for children. Hence, accuracy of measuring devices come with liquid antimicrobial agents is important not only to ensure successful treatment of infections, but also to prevent emergence of antimicrobial resistance.

We therefore, decided to develop locally acceptable standards for oral liquid measuring devices and to critically appraise the measuring devices registered in Sri Lanka using the developed standards. We also decided to verify whether the measuring devices enclosed with oral liquid antimicrobial registered in Sri Lanka are capable of measuring the volumes accurately.

## Methodology

This study was done in three stages: (1) developing the standards to assess the quality of devices intended for measuring oral liquid medicines (2) assessing the quality of measuring devices registered in Sri Lanka and (3) determining the accuracy of dose when the volume was measured by the measuring device enclosed with an oral liquid antimicrobial.

### Stage 1: Developing the standards to assess the quality of devices intended for measuring oral liquid medicines

A detailed literature search was done for ideal characteristics of a measuring device and instructions that should be available with different types of key paediatric oral liquid dosage forms.

The key questions used for developing the search strategy for the literature review were:

1. What are the quality parameters needed for liquid measuring devices?
2. What are the standards /guidelines available for liquid measuring devices?

All types of articles published in English on liquid measuring devices were eligible. Based on the research questions, the search terms, including their synonyms, truncation and spelling variants, were: “child”, “infant”,“paediatrics”, “paediatric”, “baby”, “parent”, “guardian”, “drug formulation”, “drug measuring”, “measuring”, “dosage form”, “oral liquid dosage forms”, “oral drug”, “liquids’, “syrup”, “solution”, “suspension”, “administration”, “administration devices”, ‘measuring devices’, “droppers”, “oral/enteral syringes”, “measuring cups”, “measuring spoons”, “household spoons”, “standards”, “regulations”, “guidelines”, “label”, “labelling” and “quality”. Numerous alternative search terms combined by the Boolean operators “OR” and “AND” were used to search for articles in PubMed and Scopus. “OR” widened the search and marked it highly sensitive. Using “AND” at the end of the process narrowed the search. Titles and abstracts were screened initially to identify all English-language guidelines, reports and articles on standards on oral liquid measuring devices. Duplicate studies were identified and deleted. Full-text articles were carefully reviewed to identify the articles that were aimed at answering the key questions.

First author extracted all relevant information from the articles. Based on the available literature expected standards for these measuring devices and instructions were developed. The standards were converted into a rating scale using a 9 point Likert scale (1 least to 9 most). Necessity was defined as whether the standard is an important guideline to assess the measuring devices accompanying the paediatric oral liquid dosage form. During June 2019 a multidisciplinary panel of 10 experts were selected from the area of paediatrics, pharmacology, clinical pharmacy, chemistry and regulatory pharmacy.

Ratings were entered and the median was calculated for each standard. Standards which received an overall panel median score of ≥ 7 with an agreement (no more than two panel members rating the statement outside a 3 point distribution around the median) were selected [7]. Free comments from the experts were also considered when finalising the standards. These selected standards were subjected to further securitization by the authors. Based on the final list of standards, a structured checklist was prepared.

### Stage 2: Assessing the quality of measuring devices registered in Sri Lanka

It was a descriptive cross-sectional study. Paediatric oral liquid dosage forms of medicines registered with National Medicines Regulatory Authority’s (https://nmra.gov.lk/) and available in the market for a consumer at the time of this study were purchased and their measuring devices were assessed.

#### Data collection

Two investigators independently assessed all the measuring devices, labels and instructions enclosed with these products using a checklist prepared from the standards developed in the stage one. In case of disagreement between the two reviewers, decision by consensus was taken. Inputs from 3^rd^ investigator was obtained when there was a disagreement between the two reviewers. Agreement of 2 of the 3 investigators was taken as final. Cohen’s kappa was calculated using a contingency table to determine the inter-rater agreement. A kappa value for all the standards above 0.7 was considered as satisfactory. Data were entered and analysed.

### Stage 3: Determining the accuracy of dose when the volume was measured by the measuring device enclosed with an oral liquid antimicrobial

Only the oral liquid antimicrobial suspensions were selected. Five pharmacy undergraduates were recruited for this purpose. Each of them were provided with paediatric oral liquid antimicrobial preparations currently registered and available in Sri Lanka. The powder for reconstitution was reconstituted according to the instruction provided with the product. Participants measured three volumes (5 mL, 3.75 mL and 2.5 mL) for each of the products using the enclosed measuring device. Accuracy of the volumes measured by the participants was verified by comparing the weight of the measured dose to a reference weight for 5, 3.75 and 2.5 mL using a calibrated analytical weighing balance. The reference weight for 5 mL was determined by averaging the weight of 5 mL dose measured by 5 senior pharmacists using a syringe. This was done for 2.5 ml and 3.75 ml also. United States Pharmacopeia’s definition for volume error (greater than 10%) for 5 mL liquid medicines was used in assessing the accuracy of dose^.^[8] Dosing error within ±10% was considered as no error, between ±11–20% as ‘mild error’ and > 20% as ‘moderate error’

Ethics statement: Ethical approval (EC-18-006) was obtained from the Ethics Review Committee, Faculty of Medicine, University of Colombo, Sri Lanka

## Results

### Stage 1

The final list of standards which received overall panel median score of ≥ 7 with agreement (no more than two panel members rating the statement outside a 3 point distribution around the median) are given below.

1. Dosage delivery devices should be included for all oral liquid dosage forms.
2. Calibrated units of liquid measure marked on the device should be same as the units of liquid measure specified in the labelled dosage directions.
3. The abbreviation on the device should be the same as in the labelled dose directions.
4. International or national standards for abbreviations should be used.
5. Millilitre-based dosing should include leading zeros preceding decimals for doses less than 1 mL to avoid 10-fold dosing errors.
6. Avoid trailing zeros after decimal points to avoid 10-fold dosing error.
7. Smaller font size for numerals in fractions should be used
8. Devices should not be considerably larger than the largest dose described in the labelled dosage directions and should deliver the smallest labelled dosage.
9. Liquid dosage forms should be dosed to the closest 0.1, 0.5, or 1 mL, as appropriate based on the margin for safe and effective dosing
10. Dosing to the hundredth of a millilitre should be avoided.
11. Teaspoon and tablespoon units should not be used together.
12. Liquid measuring mark should be clearly visible after product is added to the dosing device.

### Stage 2

**Fig 1:**
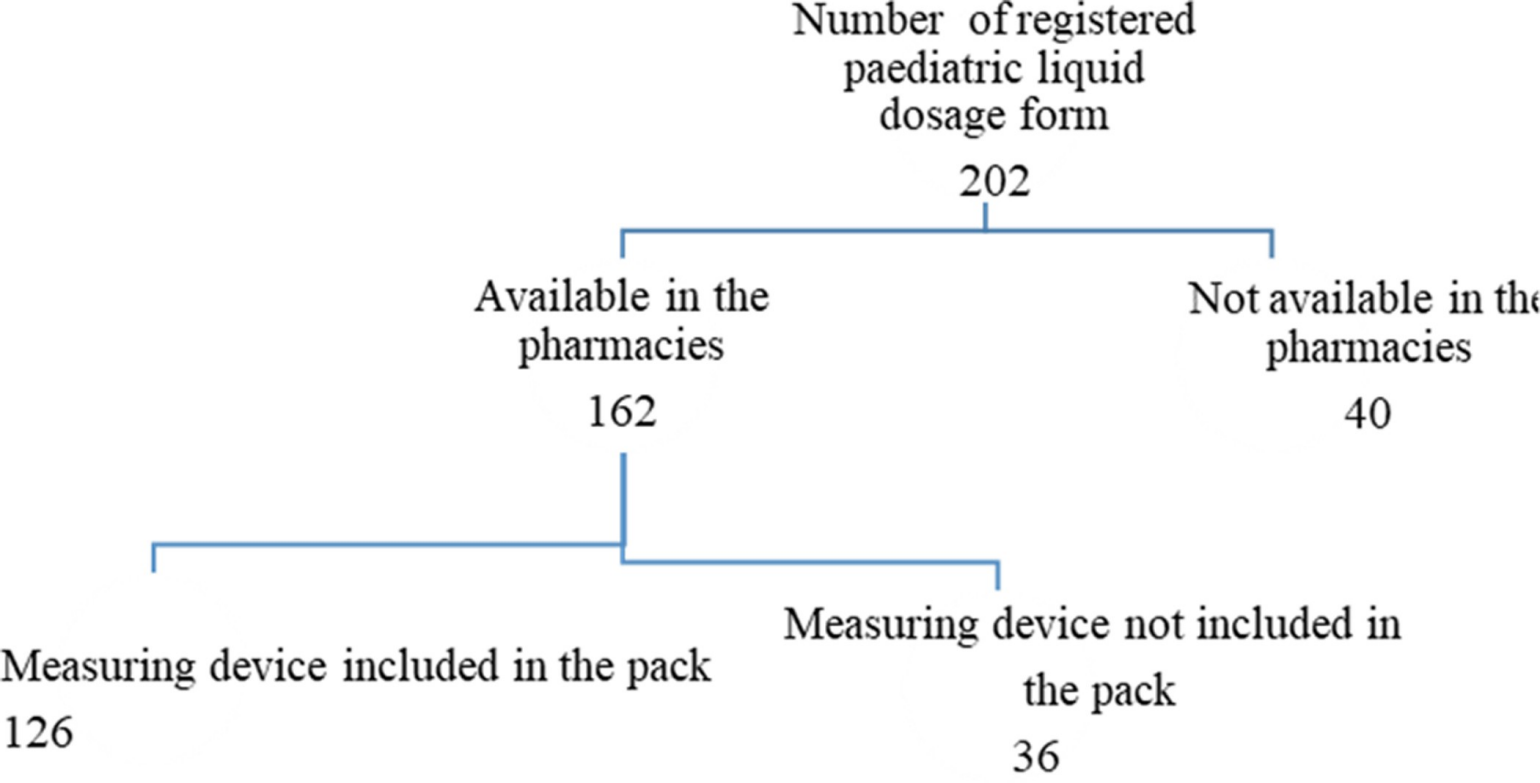
Product selection for assessing the measuring device enclosed with the oral liquid dosageform registered with NMRA.

Measuring device was not available in nearly one-fourth of the products (Fig 1). Measuring cup (83 of 126–65.9%) was the common measuring device packed with the oral paediatric dosage form followed by measuring spoon (35 of 126–27.8%). Table 1 shows the therapeutic classes of medicines and the types of measuring devices, 32.5% of anti-infective had measuring devices followed by medicines used in respiratory system (30.2%).

**Table 1:** Anatomical Therapeutic Chemical classification of oral liquid dosage forms coming with measuring devices.

ATC Classification	Type of measuring device packed					Device not available (%)	Total number of oral dosage forms (%)
	O*	C*	S*	D*	Combination*		
Alimentary tract and metabolism	00	19	03	01	00	20 (15.9)	43 (26.5)
Blood and blood forming organs	00	08	00	02	00	04 (3.2)	14 (8.6)
Anti-infective for systemic use	01	23	13	01	03	01(0.8)	42(25.9)
Musculo-skeletal system	00	03	00	00	00	02(1.6)	05(3)
Nervous system	00	04	02	00	00	00(0)	06(3.7)
Antiparasitic insecticides and repellents	00	02	00	00	00	00(0)	02(1.2)
Respiratory system	00	21	16	00	00	09(7.1)	46(28.4)
Sensory organs	00	03	01	00	00	00(0)	04(2.5)

*: C: Measuring cup; S: Measuring spoon; O: Oral Syringe; D: Dropper, combination: Two devices.

As shown in Table 2, none of the devices met all the standards. Range of adherence to individual criterion in the set of standards was 60.3–98.4%.

**Table 2:** Measuring devices which aligns with criteria of the standards developed.

No	Standard	Number of products aligning to the criteria / Total no of products *	% (95% CI)
1	Dosage delivery device included	126/162	77.8 (70.6–83.9)
2	Calibrated units of liquid measure marked on the device are the same as the units of liquid measure specified in the labelled dosage direction	76/126	60.3 (51.2–68.9)
3	Abbreviation used on the device is the same as in the labelled dosage directions	80/126	63.5 (54.4–71.9)
4	International or national standards for abbreviations is used	115/126	91.3 (84.9–95.6)
5	Millilitre-based dosing include leading zeros preceding decimals for doses less than 1 mL	121/126	96.0 (91.0–98.7)
6	No trailing zeros after decimal points	120/126	95.2 (89.9–98.2)
7	Smaller font size for numerals in fractions used	48/67	71.6 (59.3–82.0)
8	Dosage delivery devices is not significantly larger than the largest dose described in the labelled dosage directions	70/126	55.6 (46.4–64.4)
9	Smallest labelled dosage marked on the device	84/126	66.7 (57.7–74.8)
10	The device should be able to deliver the correct dose to the nearest 0.1, 0.5, or 1 mL	124/126	98.4 (94.4–99.8)
11	Dosing to the hundredth of a millilitre avoided	120/126	95.2 (89.9–98.2)
**No**	**Standard**	Number of products aligning to the criteria / Total no of products *	**%** **(95% CI)**
12	Teaspoon and tablespoon units not used together	36/43	83.7 (69.3–93.2)
13	Liquid measuring mark is be clearly visible after product is added to the dosing device	124/126	98.4 (94.4–99.8)

* No. represents the number of products that adhere the standards; Total No. represents the total number of products relevant to the standard of concern.

The kappa value for all the standards was above 0.7 which indicates a high agreement between the two raters.

In nearly 40% the calibrated units of liquid measure marked on the device were not the same as the units of liquid measure specified in the labelled dosage direction. The abbreviation used on the device was not the same abbreviation used in the labelled dosage directions. Almost half of the dosage delivery devices were significantly larger than the largest dose described in the labelled dosage directions. Almost quarter of the devices did not use smaller font size for numerals in fractions.

### Stage 3

Table 3 shows the dosing errors noticed when using the measuring devices packaged with the 44 liquid paediatric antimicrobial analysed for this study. Dosing error was less with measuring cup when compared to other measuring devices. Only 12 measuring devices (27%, 95% CI: 15%-43%) were within 10% of the 2.5 ml target volume, defined as no errors. For 3.75 ml only 48% (95% CI: 32%-63%) were within 10% of the target volume and for 5 ml it was 45.5% (95% CI: 30%-61%).

**Table 3:** Errors which occurred when measuring three different volumes with the measuring devices packed with the antimicrobial registered with NMRA.

Device*	Total [%]	Volume measured								
		2.5ml			3.75ml			5 ml		
		No error [%]	Mild error [%]	Moderate error [%]	No error [%]	Mild error [%]	Moderate error [%]	No error [%]	Mild error [%]	Moderate error [%]
**O**	1[100]	0 [0]	0 [0]	1 [100]	0 [0]	0 [0]	1 [100]	1 [100]	0 [0]	0 [0]
**C**	25[100]	9 [36]	9 [36]	7 [28]	11 [44]	9 [36]	5 [20]	11 [44]	12 [48]	2 [8]
**S**	14 [100]	2 [14]	8 [57]	4 [29]	7 [50]	4 [29]	3 [21]	8 [57]	4 [29]	2 [14]
**D**	4 [100]	1 [25]	1 [25]	2 [50]	3 [75]	1 [25]	0 [0]	0 [0]	2 [50]	2 [50]

Percentages may not sum to 100% due to rounding. C: Measuring cup; S: Measuring spoon; O: Oral Syringe; D: Dropper.

No error: Mean volume ±10%, mild error: Mean volume ±11–20%, moderate error: Mean volume >20.

* Number of device is more than the products due to combination of products in three packs.

#### Problems encountered when reconstituting the oral liquid antimicrobial

In one product, the enclosed measuring device (5 ml measuring spoon) was to be used to measure the volume of water (60 ml) required for reconstitution. The user is instructed to measure the water 12 times in order to reconstitute the product. In some products, the mark to which the water should be added were not clearly marked on the bottle or label. In handful of products due to the colour of the bottle the liquid within was not clearly visible making it difficult to check whether the powder had been dissolved completely without any clumps.

## Discussion

This is the first study in Sri Lanka to evaluate the measuring devices enclosed with paediatric oral liquid dosage forms. A comprehensive, well tested process was followed to develop the standards. These standards will benefit regulatory body, importers and manufacturing organisations. There were only a handful of studies on the standards of measuring devices and those were similar to the US FDA. Less literature may be the reason of getting similar standards as the US FDA on measuring devices.

Standardized measuring devices were not included in all the packs. This will result in parent/caregiver using non-standardised measuring device like a household spoon. It is well known that use of a kitchen spoon increases the error when used instead of a standardized device [9].

None of the measuring devices in our study sample met all the standards. Around 50% of the devices were delivering doses larger than those listed in the directions, which will increase the potential for overdosing. It has been documented that medication dosing errors can be reduced by reducing the markings on the device to relevant recommended doses for that particular medicine [10,11].

Trailing zeros were found in 5% of the devices. This could lead to 10-fold overdosing errors like interpreting “1.0” as “10” [12,13]. In 17% of the products table spoon and teaspoon had been packed together. Error in dose could occur to error in differentiating teaspoon and tablespoon. Terms like teaspoon and tablespoon favour the use of kitchen spoons, which is also associated with measurement error [14,15].

In a study, 39.4% of parents made an error in measuring doses, and those who used tablespoons or teaspoons made errors two times higher than those who used millilitre-only measuring device [16]. It has been documented that measuring with oral syringes has been associated with less dosing errors [17], only one product in study had an oral syringe as the measuring device. Errors were obviously less when measuring cups were used for larger volumes. None of the studied measuring devices had the 3.75mL marking which would have accounted for measuring the 3.75mL incorrectly.

A limitation of this study was that we used pharmacy undergraduates who are not yet parents in our study to measure the liquid medicines. On one hand, this may not reflect parents/ caregivers’ true ability to measure the dose at home and on the other hand pharmacy undergraduates are trained in chemistry lab to use various measuring devices. If they too make errors, chances of parents making error is a strong possibility.

In few antimicrobial packs instructions for reconstitution was not accurate. In one product to reconstitute the water had to be added 12 times by a measuring spoon provided. The error rate will be higher when compared to adding water in two portions.

Underlying reasons and outcomes of this study should be studied further. Regulatory body should ensure that during the registration of the medicines a measuring device is enclosed with all the pediatric oral liquid dosage forms. Standards should be developed to assess the quality of the measuring devices. Until the Sri Lankan standards are developed the regulatory authorities could use the international standards such as US FDA, EMA on measuring devices to assess the quality of measuring devices.

## Conclusion

A set of standards to check the quality of oral liquid measuring devices enclosed with the packs was developed using a well-accepted standard method. Of the registered products, 20% did not have a measuring device in the pack. More than 50% of the rest of the products complied with the standards developed. Less volume error was seen with measuring cup when compared to other measuring devices packed with liquid antimicrobial. Quality could be further improved if the regulatory body requests the manufacturers/importers to adhere to the standards developed.

Commercially available measuring devices with paediatric oral liquid dosage forms in Sri Lanka were not up to the standard expected from such a device. Over 50% of pharmacy undergraduates failed to measure the correct volume using the measuring devices provided with the liquid antimicrobial agents. This was higher with smaller volumes. Inability to administer the correct dose to children because of substandard measuring devices needs regulatory interventions.

## Supporting information

S1 Checklist STROBE statement—checklist of items that should be included in reports of observational studies. (DOCX)
